# Mutations in *COL1A1* and *COL1A2* and dental aberrations in children and adolescents with osteogenesis imperfecta – A retrospective cohort study

**DOI:** 10.1371/journal.pone.0176466

**Published:** 2017-05-12

**Authors:** Kristofer Andersson, Göran Dahllöf, Katarina Lindahl, Andreas Kindmark, Giedre Grigelioniene, Eva Åström, Barbro Malmgren

**Affiliations:** 1 Department of Dental Medicine, Division of Pediatric Dentistry, Karolinska Institutet, Huddinge, Sweden; 2 Center for Pediatric Oral Health Research, Stockholm, Sweden; 3 Department of Medical Sciences, Uppsala University, Uppsala, Sweden; 4 Department of Clinical Genetics, Karolinska University Hospital, Stockholm, Sweden; 5 Department of Molecular Medicine and Surgery, Karolinska Institutet, Stockholm, Sweden; 6 Department of Woman and Child Health, Karolinska Institutet, Stockholm, Sweden; 7 Pediatric Neurology and Musculoskeletal disorders and Home care, Astrid Lindgren Children's Hospital at Karolinska University Hospital, Stockholm, Sweden; University of North Carolina at Chapel Hill, UNITED STATES

## Abstract

Osteogenesis imperfecta (OI) is a heterogeneous group of disorders of connective tissue, caused mainly by mutations in the collagen I genes (*COL1A1* and *COL1A2*). Dentinogenesis imperfecta (DGI) and other dental aberrations are common features of OI. We investigated the association between collagen I mutations and DGI, taurodontism, and retention of permanent second molars in a retrospective cohort of 152 unrelated children and adolescents with OI. The clinical examination included radiographic evaluations. Teeth from 81 individuals were available for histopathological evaluation. *COL1A1/2* mutations were found in 104 individuals by nucleotide sequencing. DGI was diagnosed clinically and radiographically in 29% of the individuals (44/152) and through isolated histological findings in another 19% (29/152). In the individuals with a *COL1A1* mutation, 70% (7/10) of those with a glycine substitution located C-terminal of p.Gly305 exhibited DGI in both dentitions while no individual (0/7) with a mutation N-terminal of this point exhibited DGI in either dentition (p = 0.01). In the individuals with a *COL1A2* mutation, 80% (8/10) of those with a glycine substitution located C terminal of p.Gly211 exhibited DGI in both dentitions while no individual (0/5) with a mutation N-terminal of this point (p = 0.007) exhibited DGI in either dentition. DGI was restricted to the deciduous dentition in 20 individuals. Seventeen had missense mutations where glycine to serine was the most prevalent substitution (53%). Taurodontism occurred in 18% and retention of permanent second molars in 31% of the adolescents. Dental aberrations are strongly associated with qualitatively changed collagen I. The varying expressivity of DGI is related to the location of the collagen I mutation. Genotype information may be helpful in identifying individuals with OI who have an increased risk of dental aberrations.

## Introduction

Osteogenesis imperfecta (OI) is a genetic connective tissue disorder, with the cardinal symptom being bone fragility, often leading to growth retardation. Affected individuals may also have blue sclera, joint laxity, and dentinogenesis imperfecta (DGI or mentioned as DI type I). OI is traditionally classified into four main types according to clinical and radiographic findings [1], where type I is mild with blue sclerae, type II is pre- or perinatally lethal, type III is the most severe type associated with survival of the perinatal period, and type IV is of moderate severity.

Autosomal dominant mutations in the *COL1A1* and *COL1A2* genes are causative in approximately 85% of cases [2–4], but more recent studies have revealed that mutations in these genes are less frequently found in moderate to severe cases of OI [5, 6]. Collagen I is composed of two α1 chains and one α2 chain, which form a triple helix. Gly-X-Y triplets characterize the helical portion of the α1 and α2 chains, where glycine occurs in every third position, as this is the only residue that fits sterically in the center of the helix. Amino (N)- and carboxy (C)-termini flank the helical domain.

There are two main types of collagen I mutations in OI—those that lead to structurally normal, but quantitatively changed type I procollagen, and those that cause a structurally abnormal protein. Quantitative defects are generally the result of a premature termination codon in *COL1A1* [7, 8].These lead to haploinsufficiency of collagen type I, and are often seen in mild OI type I. Qualitative defects are often caused by mutations affecting the protein sequence of the triple helical domain [9]. A qualitatively changed protein is associated with a more severe skeletal phenotype [9–11].

**Fig 1:**
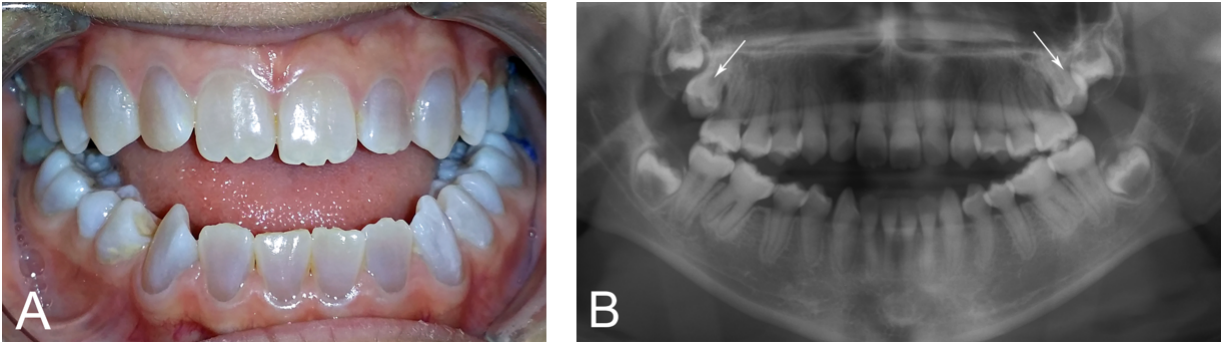
Clinical appearance of dentinogenesis imperfecta (DGI) and retention of permanent second molars. A 15-year-old girl with OI type I and DGI and a splice mutation in *COLIA2*, c.1197+5G>A. **A.** Clinical picture of DGI with characteristic grey-blue discoloration, and a Class III malocclusion with an anterior open bite. **B**. Typical signs of DGI with calcified pulp chambers and bulbous crowns with constriction at cervix. Retention of both upper permanent second molars with a vertical inclination can be noted (arrows).

DGI is a common feature in OI (Fig 1) [12, 13]. Depending on type of OI, prevalence is estimated to be between 8% and 100%. More severely affected children often have a more severe dental phenotype [12–14]. A relationship between qualitatively changed collagen I and DGI has previously been found [6, 15, 16].

Additionally, many individuals with OI present with other dental aberrations. We have previously reported a high prevalence of hypodontia and oligodontia in adolescents with OI [17]. Apically extended pulp chambers (taurodontism), primary retained permanent second molars, (failure of eruption before emergence, without a physical barrier in the eruption path) [12, 18] and aberrant craniofacial development with a retrognathic maxilla often occur in patients with OI [19, 20]. Taurodontism is a rare condition in healthy individuals which occurs in 0.3–2.5% [21, 22]. Retention of permanent second molars is an unusual finding in healthy individuals. Bondemark and Tsiopa observed retention in 0.6% of healthy adolescents [23].

This study investigated the dental phenotype of children and adolescents with OI and related it to collagen I mutations with an emphasis on presence of DGI in the deciduous or in both the deciduous and permanent dentitions, on taurodontism, and on retention of permanent second molars. Our hypotheses were that DGI in both dentitions, taurodontism, and retention of permanent second molars are more prevalent in individuals with qualitative defects caused by mutations in *COL1A1* and *COL1A2*, and that presence of dental aberrations are related to the type of OI.

## Materials and methods

Children and adolescents aged 0.4–19 years with OI and cared for at the Astrid Lindgren Children’s Hospital at Karolinska University Hospital, Stockholm (Sweden’s national multi-disciplinary pediatric OI team), between 2005 and 2014 were invited to participate in the study. Thus, this retrospective cohort study assessed 179 individuals from 152 families. To avoid skewing the genetic impact of each mutation, we included only one child per family, the child with the most complete clinical data. Any family history of DGI was noted. Children with no erupted teeth were excluded. The final study group comprised 152 individuals, 67 females and 85 males, of whom 63% (96) were classified as OI type I, 15% (22) as OI type III, and 22% (34) as OI type IV. Intravenous monthly infusion with pamidronate was given to 84 individuals of whom 66 were younger than 13 years at start of treatment. Mean age at the most recent evaluation of dental phenotype was 12.1±5.1 years (median 12.8 years; range 0.4–19 years).

The Swedish regional ethics committees in Stockholm and Uppsala approved the study protocol (Daybook no. [Dnr] 157/99, 2014/254-31 and Ups 2006/212). Written informed consent was obtained from recruited participants and their guardians.

### Mutation analysis

#### Sanger sequencing

Sanger sequencing of peripheral blood DNA was done. Polymerase chain reaction (PCR) primers were designed to cover the coding exons and flanking introns of the *COL1A1* and *COL1A2* genes. The PCRs were performed on a GeneAmp PCR system 9800 using AmpliTaq Gold kits, and the sequencing reactions according to an adjusted Big Dye Terminator 3.1 sequencing protocol, all per the manufacturers’ recommendations. Products were run on a 16-capillary ABI 3130xl Genetic Analyzer automated sequencer and analyzed with SeqScape v2.5. All reagents, equipment and software were purchased from Applied Biosystems, CA, USA (www.appliedbiosystems.com). All identified variants were confirmed by resequencing the afflicted exon and, when necessary, conducting a segregation analysis in family members.

#### Multiplex ligation-dependent probe amplification

29 individuals found to be negative for mutations after Sanger sequencing underwent multiplex ligation-dependent probe amplification (MLPA) analysis of *COL1A1* and *COL1A2* in order to detect large deletions and insertions. We used SALSA-MLPA Kits P271 *COL1A1* and P272 *COL1A2* (MRC-Holland, Holland) according to the manufacturer’s instructions (www.mlpa.com).

#### Nomenclature

We followed the recommendations of the Nomenclature Committee of the Human Genome Variation Society (www.HGVS.org/varnomen) to describe sequence variations and used the GenBank reference sequences of *COL1A1* (genomic DNA NG_007400.1 and cDNA NM_000088.3) and *COL1A2* (genomic DNA NG_007405.1 and cDNA NM_000089.3).

Mutations were reported to NCBI ClinVar (https://www.ncbi.nlm.nih.gov/clinvar/), the osteogenesis imperfecta & Ehlers-Danlos syndrome variant database (http://www.le.ac.uk/genetics/collagen/) [24, 25] and have previously been described [6, 26].

### Clinical, radiographic, and histological examinations

All dental examinations had been done at Astrid Lindgren Children’s Hospital, Eastman Institute, or Karolinska Institutet in Stockholm between 1992 and 2015. The clinical examination included recording signs of DGI. Characteristic grey-blue or yellow-brown discoloration of deciduous or permanent tooth crowns solely or in combination with pathological attrition and/or fractures were regarded as clinical signs of DGI. The diagnosis of DGI was based on a combination of clinical, radiographic, and/or histological findings. Photographic documentation was done. Two authors (KA and BM) retrospectively evaluated clinical and radiographic status.

96 teeth, 23 permanent and 73 deciduous, from 81/152 patients were available for histopathologic analysis. The permanent teeth had been extracted for orthodontic or other therapeutic reasons, while most deciduous teeth had been exfoliated. We prepared the teeth as previously described [27] and assessed ground sections for dysplastic manifestations in the dentin. Such manifestations included branching of dentin tubules, variations in the width of the dentin tubules, presence of hyaline dentin void of dentin tubules, layering of dentin, and presence of cell lacunae and duct-like structures in the body of the dentin.

Panoramic radiographs were available for 123 individuals. Only bite-wing or periapical radiographs were available for 13 children, and no radiographic examination had been done in 16 individuals due to their young age (<4 years of age). The radiographic evaluation of DGI included recording abnormalities in crown shape, cervical constrictions, and abnormally large or calcified pulp chambers. We assessed the lower first permanent molars for signs of taurodontism since we judged these teeth would yield the most reliable assessment based on radiographic projection. The degree of the relative amount of apical displacement was based on the size of the pulp chamber and the size and shape of the roots [28]. We excluded individuals with radiographic signs of DGI in the permanent dentition because taurodontism is impossible to assess in teeth exhibiting radiographic DGI characteristics.

We judged permanent second molars to be retained based on radiographic signs in combination with clinical findings or if they were unerupted at age 15 years. Inclination of retained second molars was assessed as mesioangular, vertical, or distoangular.

**Fig 2:**
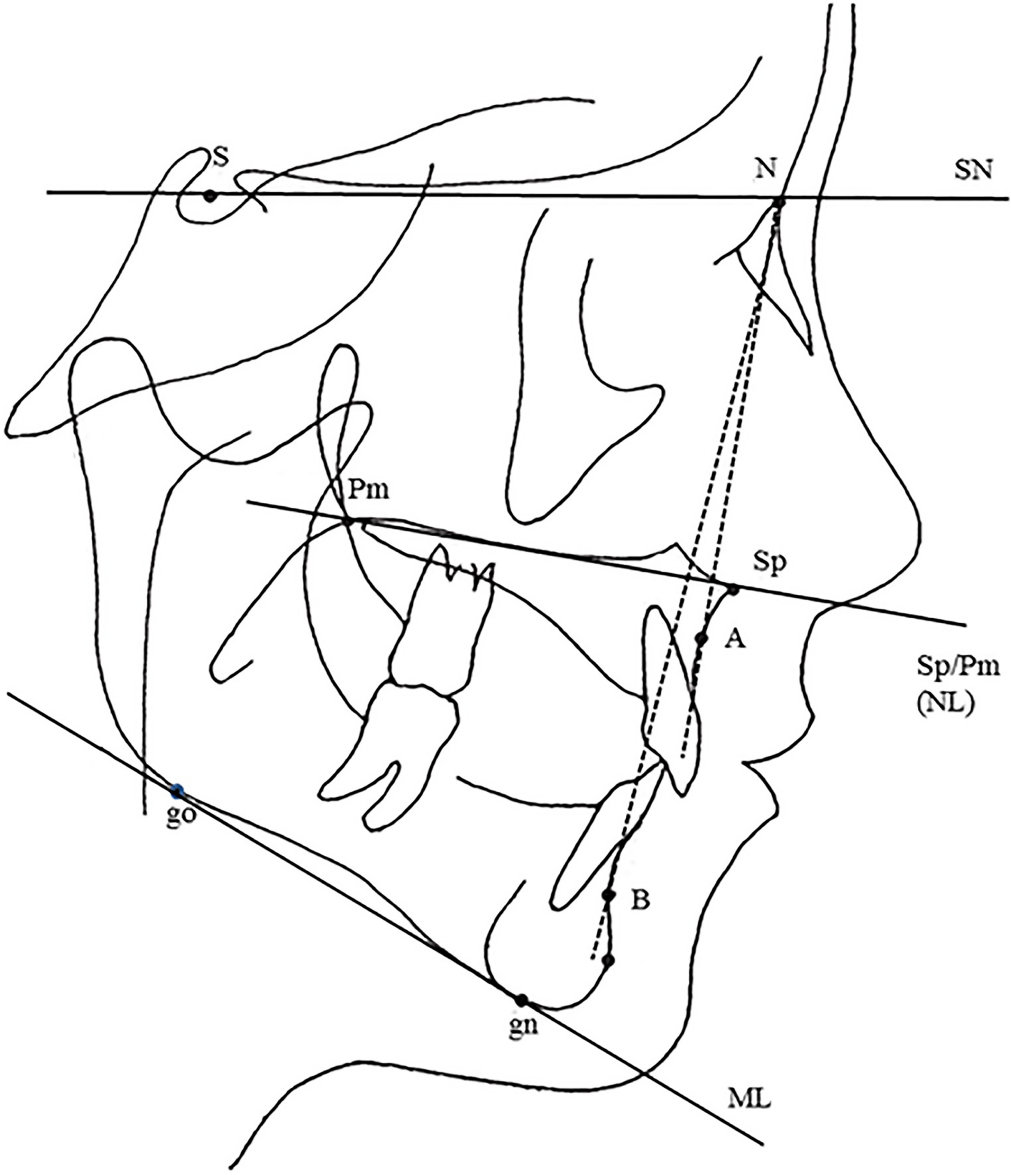
Skeletal reference points, lines and angles. Reference points: S: The center of sella turcica, N: Nasion, Sp: Spina, the apex of the anterior nasal spine, Pm: Pterygomaxillare, A: The most concave point of anterior maxilla, B: The most concave point on mandibular symphysis, Gn: Gnathion, the lowest point in the lower border of the mandible in the median plane, Go: Gonion, the most posterior inferior point on angle of mandible. Reference lines: SN, SpPm (NL), ML: The mandibular line. Reference angles: SNA, SNB, SN/SpPm, SN/ML.

Lateral cephalometric radiographs were available for 41 individuals, aged 10–19 years, 23 boys and 18 girls. These were used to relate retention of second molars to the craniofacial position of the jaws, type of OI, presence or absence of DGI, and type of mutation. Facad® software for orthodontic tracing and cephalometric analysis (ILEXIS AB, Linköping, Sweden) was used to analyze the lateral cephalograms. The positions of the maxilla and mandible were compared to normative cephalometric data for a Swedish population [29]. Normative data comprise age and gender values for healthy children with a normal occlusion. The sella–nasion–A point (SNA) angle was used to determine the position of the maxilla in the anterior-posterior direction, and the SN/SpPm angle to determine the position of the maxilla in the vertical direction. The sella–nasion–B point (SNB) angle was used to determine the anterior-posterior position of the mandible. The inclination of the mandible was determined by plotting the angle between the mandibular line (ML) and the SN line. We analyzed the inclination of the upper and lower incisors with the long axis of the teeth to the SN line and the ML (Fig 2).

### Statistical analysis

We used the *X*^2^-test for categorical variables to determine differences in frequencies of DGI and other dental aberrations. Two-tailed p-values were computed using p<0.05 to denote a significant deviation from the null hypothesis. The independent *t*-test evaluated continuous variables. We compared the value of each cephalometric measurement with the corresponding value in the normal material matched according to age and gender and calculated differences with this formula: Dev = (*X*i-$\bar{X}$ /sd) where Dev = difference, Xi = actual value, $\bar{X}$ = average value of the normal material, and sd = standard deviation of the reference material. Mann-Whitney U test was used to evaluate the differences between permanent second molar retention, cephalometric values, DGI and pamidronate treatment. All statistical analyses were done using the Statistical Package for the Social Sciences (SPSS for Windows, v. 24; IBM SPSS Inc., Chicago, IL, USA).

## Results

### Mutations in *COL1A1* and *COL1A2*

We found *COL1A1* and *COL1A2* mutations in 81% of the individuals with OI (123/152; 121 by Sanger sequencing and 2 by MLPA). Among these mutations, 54% (66/123) were assessed as causing a qualitative defect and 46% (56/123) a quantitative. In one individual presenting with a variant of unknown clinical significance, it was not possible to assess the effect on the protein. The mutations have previously been described in detail [6]. All patients were heterozygous for the collagen mutation in question. Most of qualitatively changed protein was caused by missense mutations with substitutions for glycine in the triple helical domain. The remaining mutations were splice mutations or other missense mutations that were predicted to result in a structurally abnormal protein product. The most common substitution was glycine to serine. We found no collagen mutation in 19% (29/152) of the children and adolescents. S1 Table presents details regarding type of mutation. Presence of DGI was seen in 70% (46/66) of those with a qualitatively changed protein compared to 27% (15/56) in whom a quantitative defect had been found (p<0.001).

### Dentinogenesis imperfecta

29% of the individuals (44/152) had a clinical and radiographic diagnosis of DGI and another 19% (29) only a histological diagnosis. In these 73 individuals, the prevalence of DGI was highest in children with OI type III and lowest in children with OI type I (86% vs. 31%; p<0.001; Table 1). From clinical, radiological, and histological characteristics, we distinguished three main groups of patients with DGI: 1 = clear and distinct DGI in the deciduous dentition only, 2 = DGI in both dentitions, and 3 = DGI diagnosis possible only on histological examination.

**Table 1:** Distribution of dentinogenesis imperfecta (DGI) in 152 individuals with different types of OI.

			DGI		
OI type	Gender F/M	Prevalence of DGI	Clear signs in 1^st^ dentition only	Clear signs in both dentitions	Histological signs only
I	36/60	30/96 (31%)*	7	6	16
III	15/7	19/22 (86%)*	4	9	5
IV	16/18	24/34 (71%)**	9	5	8
Total	67/85	73/152 (48%)***	20	20	29

* One individual

** two individuals, and

*** four individuals presenting with DGI in the deciduous dentition had no data from the permanent dentition due to low age.

#### Group 1—Presence of DGI in the deciduous dentition only

**Fig 3:**
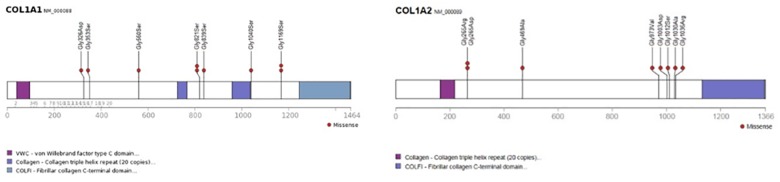
Location of mutations in *COL1A1* and *COL1A2* in individuals with clear signs of dentinogenesis imperfecta (DGI) in the deciduous dentition with no or subtle signs in the permanent dentition. Distribution of missense mutations, from N- to C-terminal, resulting in glycine substitutions in *COL1A1* and presence of DGI in the deciduous dentition with only subtle signs in the permanent dentition (n = 9). Affected residues are numbered from translation initiation. In one child, a splice mutation was found in intron 47 (c.3424-6C>G) and in one child with no signs of DGI in the permanent dentition, a nonsense mutation, p.(Ala327*), (c.972_978dup) was detected. Distribution of missense mutations, from N- to C-terminal, resulting in glycine substitutions in *COL1A2* and presence of DGI in the deciduous dentition with only subtle signs in the permanent dentition (n = 7) and no signs in the permanent dentition (n = 1). Affected residues are numbered from translation initiation.

**Fig 4:**
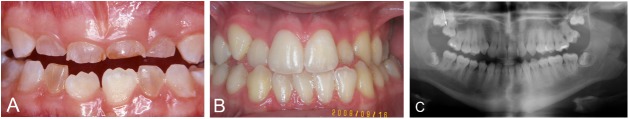
Varying expressivity of dentinogenesis imperfecta (DGI) in deciduous and permanent dentitions. A boy with OI type IV due to a missense mutation in *COLIA1*, p.(Gly821Ser), c.2461G>A, and differences in expressivity of DGI between the dentitions. **A.** At seven years of age. Discoloration and attrition in the deciduous teeth can be seen while the erupting permanent first lower incisors show no signs of DGI. **B and C.** At 16 years of age. **B.** No clinical signs of DGI are seen. **C**. Panoramic radiograph showing retention with a mesioangular inclination of the right upper permanent second molar (arrow). Only discreet signs of DGI are seen.

20 individuals exhibited DGI in the deciduous dentition only. Of these, 3 exhibited clinical, radiographic, and histological signs of DGI in the deciduous dentition only with no findings in the permanent dentition. In the other 17, however, DGI was diagnosed clinically and radiographically in the deciduous dentition with only subtle clinical signs in the permanent dentition (Fig 3). These signs included slight discoloration of the mandibular incisors. In some cases, the diagnosis could be confirmed only by radiographic examination (Fig 4). 17 individuals had missense mutations where the most prevalent substitution was glycine to serine (53%) of the missense mutations. In one child, no mutation could be detected.

#### Group 2—Presence of DGI in both dentitions

**Fig 5:**
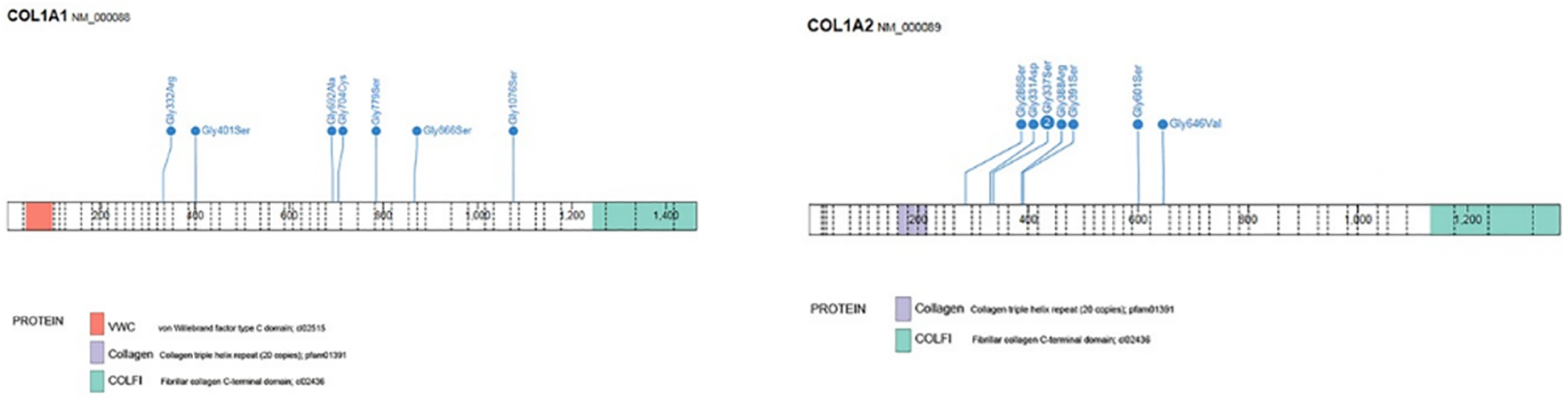
Location of mutations in *COL1A1* and *COL1A2* in individuals with dentinogenesis imperfecta (DGI) in both dentitions. Distribution of missense mutations, from N- to C-terminal, resulting in glycine substitutions in *COL1A1* and presence of DGI in the deciduous and permanent dentition (n = 7). In two children mutations were found in intron 32 (c.2235+1G>A); and in one in intron 21 (c.1197+5G>A). Distribution of missense mutations, from N- to C-terminal, resulting in glycine substitutions in *COL1A2* and presence of DGI in the deciduous and permanent dentition (n = 8). In one individual a mutation was found in intron 43 (c.2835+1G>A). Affected residues are numbered from translation initiation.

29% (20/69) of the individuals for whom data on both dentitions were available had clear and distinct signs of DGI. Of these, 70% (7/10) had a glycine substitution located C-terminal of p.Gly305 in *COL1A1*, and 80% (8/10) had a glycine substitution located C-terminal of p.Gly211 in *COL1A*2. Four children harbored other mutations predicted to cause a qualitative defect, and in 1 case, we found no collagen I mutation (Fig 5).

Significant between-group differences occurred, depending on the localization of the glycine substitution. Among group-2 individuals with *COL1A1* mutations who had a glycine substitution C-terminal of p.Gly305, 70% (7/10) exhibited DGI in both dentitions compared to no group-2 individual (0/7) with a mutation N-terminal of this point (p = 0.001). No individual with an N-terminal mutation exhibited clinical or radiographic signs of DGI in their deciduous teeth.

In individuals with a glycine substitution located C-terminal of p.Gly211 in *COL1A2*, 80% (8/10) exhibited DGI compared to no individual (0/5) presenting with a mutation N-terminal of this point (p = 0.007). None of the individuals with an N-terminal mutation exhibited any signs of DGI in their deciduous dentition, but in these patients, histological sections were not available. The congruence between presence of clinical DGI in the deciduous dentition and presence of DGI in the permanent dentition was high in all types of OI. In children with OI type III and clinical signs of DGI in their deciduous teeth, 90% (9/10) exhibited clinical DGI in their permanent successors.

#### Group 3—presence of isolated histological DGI

**Fig 6:**
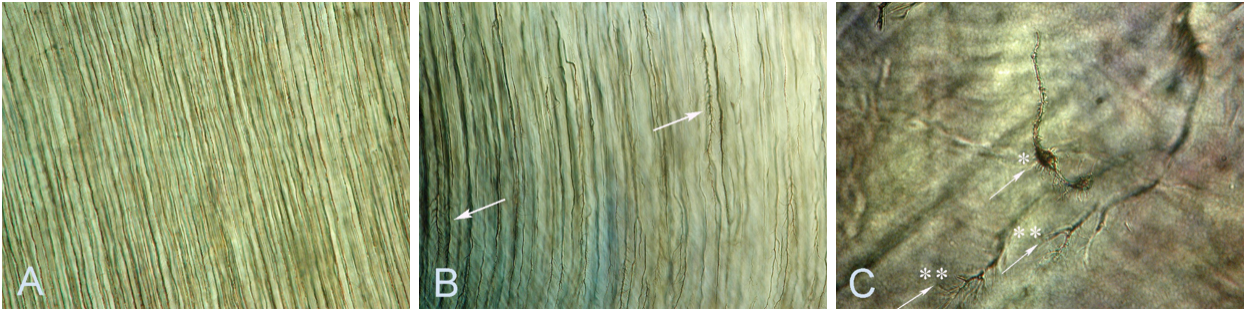
Histological findings in individuals with varying expressivity of dentinogenesis imperfecta (DGI). **A.** Ground section illustrating normal dentin. **B.** Ground section of a lower deciduous molar from an individual with OI type I and clinical DGI only in the deciduous dentition. Variation in width and branching of some dentin tubuli (arrows). No cell lacunae in the body of the dentin or layering of dentin are seen. **C.** Ground section of a lower permanent molar from an individual with OI type III and DGI. Cell lacunae* and branching of dentin tubuli** (arrows). The extent of hyaline dentin void of dentin tubuli is extensive compared to A and B.

Ground sections were available for 54 individuals with no clinical or radiographic signs of DGI, and for 27 of the individuals in groups 1 and 2 with clinical and radiographic DGI. In individuals without clinical or radiographic signs of DGI, a diagnosis of DGI was confirmed histologically in 54% (29/54) of the cases. The most frequent finding was dysplastic dentin with branching and variation in the width of the dentin tubules. The histological changes were milder compared to those of the patients with clinical and radiographic signs of DGI (Fig 6). Quantitatively changed protein occurred in 45% and frameshift mutations were most prevalent. The risk of harboring isolated histological DGI was significantly higher among those with a quantitative defect compared with individuals with a qualitative, 54% (13/24) vs. 16% (6/38; p = 0.002). No individual with isolated histological DGI in the deciduous dentition exhibited signs of DGI clinically or radiographically in the permanent dentition.

### Taurodontism

**Fig 7:**
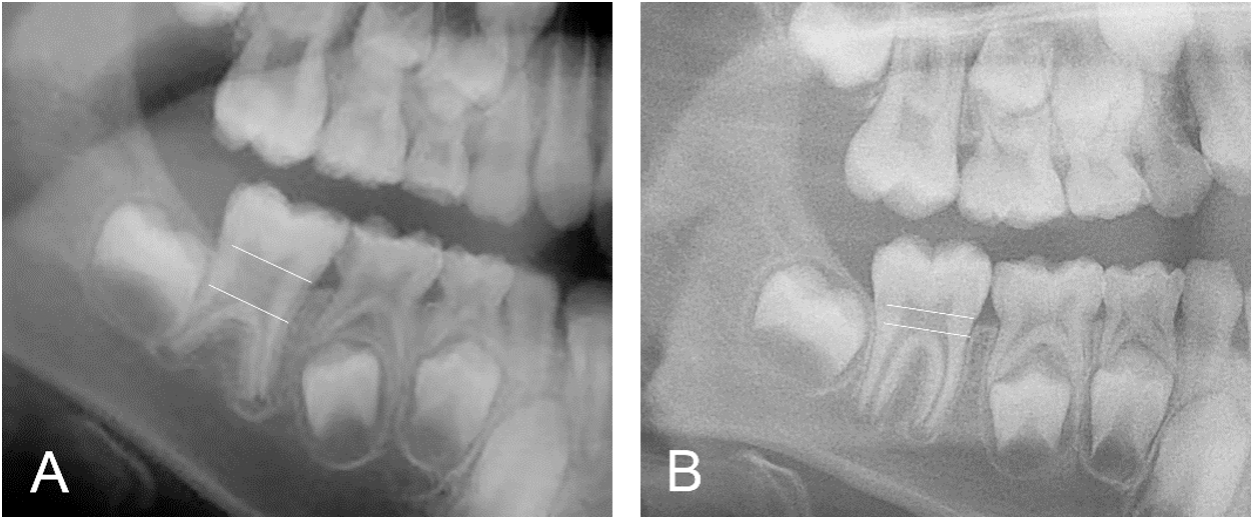
Radiographic appearance of taurodontism. Two eight-year-old boys with OI type I, quantitative defects caused by a mutation in *COL1A1* and no DGI. **A.** In this individual, apically extended pulp chamber, taurodontism, in the right first permanent lower molar is seen. **B.** Normal morphology of the corresponding tooth in the other boy.

**Fig 8:**
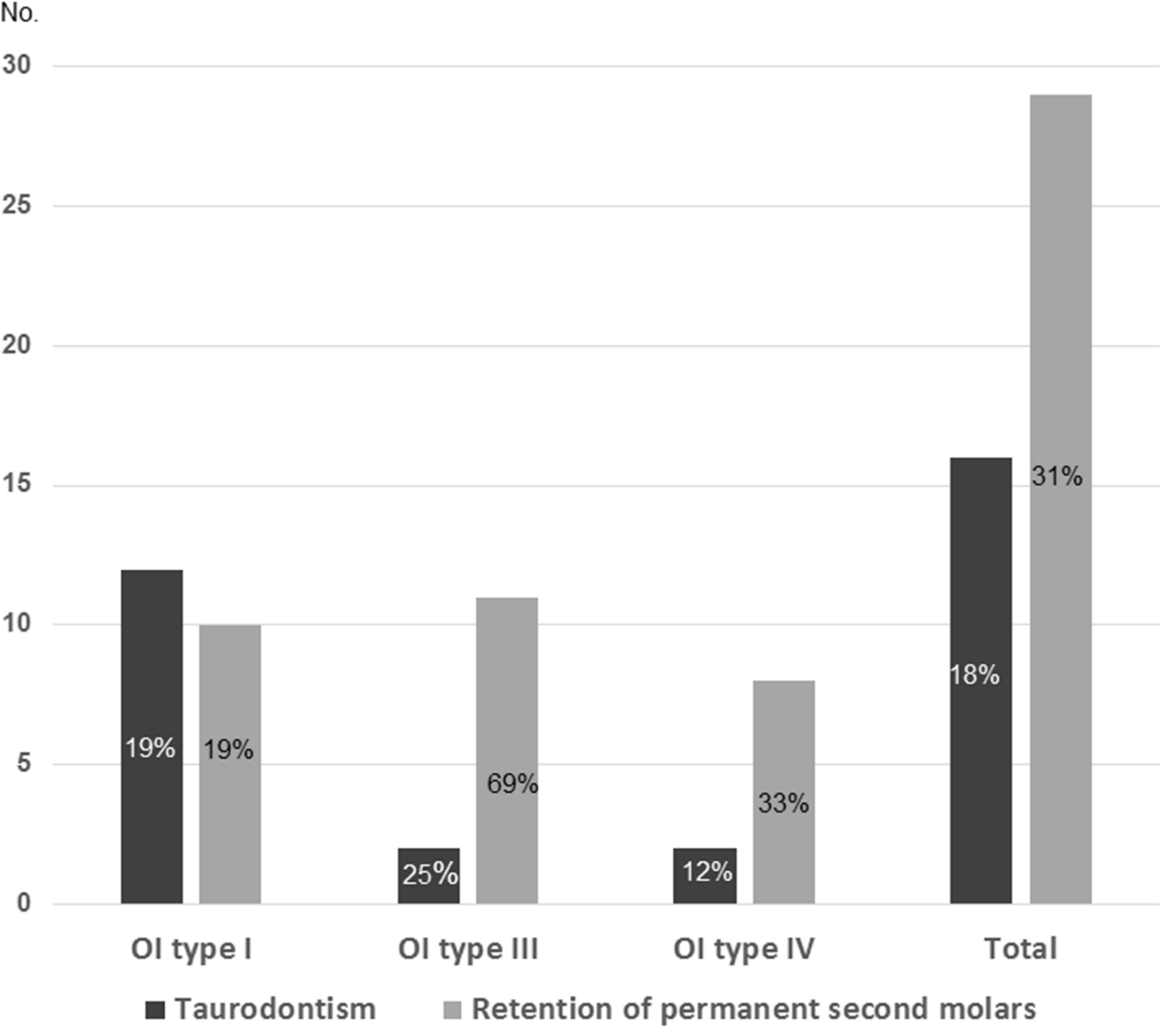
Distribution of taurodontism and retained permanent second molars in different types of OI. Taurodontism could be evaluated in 87 individuals and retention of permanent second molars in 93.

Taurodontism occurred in 18% (16) (Fig 7) of the 87 individuals who could be evaluated for this condition (Fig 8). The highest frequency occurred in individuals with no collagen I mutation, 23% (5/22). There was no significant difference in occurrence of taurodontism between individuals with quantitative 18% (7/40) and individuals with qualitative 13% (3/24) defects. Taurodontism also occurred in the boy presenting with a collagen I variant of unknown clinical significance. Hypotaurodont teeth (mild taurodontism) were most common. 78% (25/32) teeth were classified as hypotaurodont, 16% (5/32) as mesotaurodont (moderate). In two individuals unilateral cynodont (normal) teeth were found, 6% (2/32) while no teeth were hypertaurodont (severe).

### Retention of permanent second molars

31% (29/93) exhibited retention of permanent second molars (Figs 1, 4 and 8). Of the evaluated individuals, 16% (5/32) with a quantitative defect exhibited retention compared to 50% (21/42) of those with a qualitative defect (p = 0.003). The most frequent qualitative defect was caused by a missense mutation with glycine to serine substitution. 69% of the individuals with OI type III and 19% of those with OI type I (p = 0.001; Fig 8) exhibited retention. DGI in the permanent dentition occurred in 55% (16/29) of the adolescents. Number of retained second molars varied between 1 and 4 (mean 2.3±1.2; median 2.0). The more common inclination was maxillary mesioangular retention, 63% (27/43), while the corresponding figure in the mandible was 46% (11/24). Molar retention in the individuals with OI differed significantly from the Swedish reference population. Retention of permanent second molars was found in 17 of the 41 individuals in whom cephalometric analysis could be performed. Retention of second molars was found in 15 patients in the upper jaw, in six in both jaws and in two only in the lower jaw. Significantly larger values were found for the angle SN to SpPm for patients with retention in the upper jaw (p = 0.017) and for those with retention in both jaws (p = 0.003) compared to individuals in the reference population. Significantly lower values were found for the angle ML to SpPm for patients with retention in the upper jaw (p = 0.044). No significant difference between individuals with OI and the reference population was found in the SpPm angle and ML. Pamidronate treatment or presence of DGI had no significant effect on retention of permanent second molars.

## Discussion

We analyzed the association between collagen I mutation type and DGI, taurodontism, and retained permanent second molars in children and adolescents with OI. We found genetic differences between individuals exhibiting DGI in the deciduous dentition only and those exhibiting DGI in both dentitions.

In this cohort, the prevalence of DGI was 29%, which agrees with Lund *et al*.[15], but is lower than in other studies [12–14, 30]. The variation in reported prevalence is probably due to differences in the study groups and in the diagnostic methods. DGI affects children and adolescents with OI types III and IV more often than with type I; subsequently, comparisons between studies may be challenging if study group composition of OI type varies. Thus, contrary to previous studies [14, 15, 18, 30], we decided to include only one child per family to avoid skewing the genetic impact of each mutation. Other plausible explanations for variations in reported prevalence include cohort size, percentage of radiographically evaluated individuals, and age of the individuals at assessment. Based on our findings, individuals with no signs of DGI in the permanent dentition may have had DGI in their deciduous dentition. When we included clinically unaffected individuals with only histological signs of DGI, the prevalence in our population increased to 48%.

Why DGI only occurs in the deciduous dentition of some individuals remains to be elucidated. One can speculate that epigenetic space- and time-specific factors have a crucial role. These include the relative short time span for development of the deciduous teeth compared to the permanent successors, which may increase the susceptibility to developmental disturbances induced by mutations in genes related to collagen I. A possible clinical explanation for the more affected deciduous dentition and the permanent mandibular incisors is the thinner enamel in these teeth, increasing the translucency of the discolored dentin. It has previously been found that the expression of pro-alpha 1(I) and pro-alpha 2(I) may alternate during dental development [31, 32]. Intense expression of pro-alpha 2(1) has been found previously in extracted permanent teeth [33] and in mouse teeth [31]. However we found a similar frequency of missense mutations in *COL1A1* and *COL1A2* in individuals with clear signs of DGI in the deciduous dentition with no or only subtle signs in the permanent dentition. Most of the children and adolescents with a missense mutation in *COL1A2* and distinct signs of DGI only in their deciduous dentition had a missense mutation located C-terminal of p.Gly469 while most individuals with clear signs in both dentitions presented with a glycine substitution located between p.Gly286 and p.Gly391. The mutations in *COL1A1* were more equally distributed along the chain. In individuals with regional findings of mutations in *COL1A2*, the presence of Gly-Ser substitutions were less common compared to individuals with a *COL1A1* or *COL1A2* mutation who presented with clear signs of DGI in both dentitions. Clinical variability between and within different types of OI and between nonrelated individuals with the same mutation is extensive. However, the inter-individual and intra-familial DGI phenotype is often more congruent compared to other clinical outcomes [16, 34].

Pamidronate treatment had no effect on severity of DGI in the permanent dentition. Our findings are not surprising. The composition of bone and dentin is similar, but there are fundamental physiologic differences. No osteoclasts are present in dentin and no continuous remodeling is seen. The positive effects of bisphosphonates on bone tissue are not applicable to dentin.

We found that mutations causing a qualitative collagen I defect are associated with an increased risk of developing clear signs of DGI in both dentitions. The risk was higher in individuals with a glycine substitution located C-terminal of p.Gly305 and p.Gly211 in *COL1A1* and *COL1A2*, respectively. The C- versus N-terminal cut-off points in this study were chosen based both on the observed distributions, and to enable comparisons with earlier studies [6, 16]. However, in contrast to these studies a distinction was made regarding presence of DGI in the deciduous only or in both dentitions. In the present study, the prevalence of DGI was slightly lower in those individuals with *COL1A1* and *COL1A2* mutations compared to previous reports [6, 16]. Frequencies reported here are not identical to those in our previous publication [6], where all affected family members were included. Furthermore, radiographic assessments are performed continuously and added to the database. Based on our findings, children with glycine substitutions C-terminal of the above localization and presenting with clear signs of DGI in the deciduous dentition have an increased risk of DGI in the permanent dentition. Regular dental visits are essential in order to avoid later extensive treatment. Documentation with photo may be helpful in order to identify attrition over time. Functional and esthetic complaints can be successfully treated with orthodontic, restorative and prosthodontic treatment.

DGI was diagnosed histologically in 54% in a subset of individuals without clinical or radiographic signs of DGI (group 3). A majority of these individuals had quantitative defects caused by mutations in *COL1A1*. In a previous study [27], 47% of the patients with OI without clinical or radiographic DGI showed dysplastic dentin. It is possible that the prevalence of DGI in the present study would be different if more ground sections had been available. Quantitatively changed collagen I often induce a milder phenotype in the skeletal system [7, 8]. We found that a majority of the children with OI types III or IV without DGI had splice, nonsense, or missense mutations involving another amino acid than glycine or presented with unknown mutations. Our findings stress the importance of histological evaluation of individuals with suspected or confirmed OI in whom no clinical or radiographic findings of DGI are made in order to obtain a correct diagnosis. In Sweden, individuals with a confirmed DGI diagnosis are entitled to lifelong subsidized dental care as adults. This applies also to individuals presenting with isolated histological findings. Furthermore, these histological findings might be crucial in cases where the OI diagnosis is uncertain.

We have previously reported that no individuals with OI without collagen I mutation had clinical signs of DGI [6], and the present study confirms that clinical, radiographic or histological findings of DGI in both dentitions is uncommon in patients without collagen mutation. In our study cohort, only two individuals with clinical DGI had no collagen mutation: one boy with OI type III had clinical and radiological DGI in the permanent dentition, and another boy with OI type IV had clinical and radiographic signs of DGI in the deciduous dentition, but not in the permanent. In the other cases, histological examination was mandatory for confirming a diagnosis of DGI in individuals without mutation in *COL1A1* or *COL1A2*.

Taurodontism was a frequent finding in this cohort. A prevalence of 18% was high compared to a prevalence of 0.3–2.5% in the general population [21, 22]. However, the prevalence in this study was lower than in the Malmgren and Norgren study [12], which reported a prevalence of 42%. In the present study we decided to only evaluate taurodontism in mandibular permanent first molars, as this tooth was most reliably assessed based on the radiographic projection. It is reasonable to expect that the frequency would have been higher if all permanent molars had been included. Whilst taurodontism appears most frequently as an isolated anomaly, its association with other syndromes and abnormalities has also been reported, such as with Downs syndrome [35], tricho-dento-osseous syndrome [36], Williams syndrome [37], and Klinefelter syndrome [38]. It has been proposed that taurodontism has a genetic component [39]; however, because we found no correlation between taurodontism and any type of collagen I mutation, we hypothesize that the cause is not related to a specific type of collagen I abnormality. Nevertheless, our findings indicate the importance of collagen I as one of multiple components involved in the intricate epithelial-mesenchymal interactions in the morphologic development of permanent teeth. Children with OI and taurodontism did only occasionally exhibit the more severe form of taurodontism in contrast to several other syndromes. Subjective complaints are rare, however presence of taurodontism can be challenging in cases where endodontic treatment is necessary.

The high prevalence of retained permanent second molars was striking in this cohort (31%). A majority were located in the maxilla and the prevalence of DGI in these cases was similar compared to an earlier study [12]. Impaction of permanent second molars is an uncommon finding in healthy individuals (0–2.3%) [23, 40, 41]. In the present cohort, mesioangular maxillary retention was most frequent. This finding is in contrast to what is seen in healthy individuals in whom mandibular retention is most common. Failures in the eruption mechanism, physical obstacles, and crowding have been proposed etiological factors for retention of permanent second molars [42]. In this study we found that presence of one or multiple retained permanent second molars was significantly associated with a qualitatively changed protein. In individuals with OI, craniofacial characteristics differed between those with and without permanent second molar retention: in cases of retention, values for SN and SpPm were significantly larger compared to individuals in the reference population. These findings indicate that in the upper jaw, retention may be caused by lack of space due to a posterior rotation of the maxilla affecting the craniofacial development. Cephalometric evaluation shows that patients with molar retention have a reduced forward position with a clockwise rotated maxilla compared to controls. Thus retention is not primarily caused by a physical barrier due to the marked cervical constriction of the first permanent molar in individuals with DGI. Aberrant craniofacial development in OI, with a retrognathic maxilla, has been found in previous studies [19, 20]. Class III malocclusion (mandibular prognathism) was also common in this cohort. The position of the mandible was more anteriorly rotated. Inhibition of maxillary growth, maxillary hypoplasia in anteroposterior and vertical dimensions, and mandibular protrusion and hyperplasia are all possible explanations to the frequent Class III malocclusion [43–45]. A skeletal Class III malocclusion has been associated with single-nucleotide polymorphisms in *COL1A1* [46]. Our study adds to the literature that the craniofacial growth pattern may also have a negative effect on the risk of permanent second molar retention. Normal age for eruption of permanent second molars is 12–13 years. Based on this we hypothesized that start of pamidronate infusions after 13 years of age should have no effect on retention. We found no negative effect of pamidronate treatment on retention in this cohort. Treatment with intravenous bisphosphonates has been used since 1991 in individuals with severe forms of OI [47–49]. Bisphosphonates inhibit osteoclasts and bone resorption which can lead to an increased bone mass and density [50]. Although osteoclasts are important for tooth eruption the bisphosphonates have been found to have no elevated risk of abnormalities in the eruption of the permanent dentition.

Neither previous orthodontic treatment could explain the presence of retention. Subjective complaints were not frequent in our patients with retained permanent second molars. Nevertheless early diagnosis is important in order to direct development of the occlusion positively. Failure to detect retention may occasionally cause root resorption of the permanent first molar.

This cohort presents genetic findings and dental aberrations in children and adolescents with OI. All patients have been assessed at the same national center. No reported patient related outcomes and the retrospective design are possible limitations of this study. Consequently information on later complications in adulthood is scarce. The limited number of repeated mutations is another question that points at the need of more genotype-phenotype studies with an odontological approach. In conclusion, our results indicate an increased risk of developing multiple/numerous dental aberrations including DGI, taurodontism, retention of permanent second molars, and craniofacial developmental disturbances in children and adolescents with OI. The presence of DGI, and retention of permanent second molars due to deviating craniofacial development is strongly associated with qualitatively changed protein. Collagen chain mutation position is correlated to the risk of developing DGI in both dentitions. Our findings can be useful in clinical consultations to better answer questions from worried parents regarding whether their child presenting with DGI in the deciduous dentition also has an increased risk of getting an affected permanent dentition. Based on the findings in this study, taurodontism and retained permanent molars are clinical signs that support further clinical investigation to rule out a mild form of OI in an individual that is fracture prone. Finally, our results highlight the importance of carefully assessing and following children and adolescents with OI clinically, radiographically, and histologically over time in order to obtain correct diagnoses and earlier identify potential oral complications. Genetic analysis can be helpful in identifying individuals with an increased risk of oral complications.

## Supporting information

S1 Table Clinical and genetic findings in the 152 individuals with OI. Data and material (n = 152). Sequence changes [described using HGVS Sequence Variant Nomenclature and on the basis of the cDNA reference sequences NM_000088.3 (*COL1A1*) and NM_000089.3 (*COL1A2*). The dataset generated during the current study is available in the Dryad Digital Repository (https://datadryad.org/), DOI: http://dx.doi.org/10.5061/dryad.bp20k.
(DOCX)
