# Supplementary material for: Mutations in COL1A1 and COL1A2 and dental aberrations in children and adolescents with osteogenesis imperfecta – A retrospective cohort study
Source: PLoS One. 2017 May 12;12(5):e0176466. doi: 10.1371/journal.pone.0176466 (PMC5428910; doi:10.1371/journal.pone.0176466)
Supplement: S1 Table — Data and material (n = 152). Sequence changes [described using HGVS Sequence Variant Nomenclature and on the basis of the cDNA reference sequences NM_000088.3 (COL1A1) and NM_000089.3 (COL1A2). The dataset generated during the current study is available in the Dryad Digital Repository (https://datadryad.org/), DOI: http://dx.doi.org/10.5061/dryad.bp20k. (DOCX) [file pone.0176466.s001.docx]

**S1 Table. Clinical and genetic findings in the 152 individuals with OI.** Sequence changes [described using HGVS Sequence Variant Nomenclature and on the basis of the cDNA reference sequences NM_000088.3 (*COL1A1*) and NM_000089.3 (*COL1A2*).

| **Gender** | **OI type (I-IV)** | **DGI = 1 No DGI = 0** | **Gene** | **Affected Exon** | **Predicted protein** | **Sequence changes** | **Mutation Qualit. = 1 Quant. = 0** | **Type of mutation** |
| --- | --- | --- | --- | --- | --- | --- | --- | --- |
|  |  |  |  |  |  |  |  |  |
| M | I | 1 | *COL1A1* | 19 | p.(Gly431Valfs*110) | c.1292delG | 0 | Frameshift |
| M | I | 0 | *COL1A1* | 19 | p.(Gly424Alafs*117) | c.1269delC | 0 | Frameshift |
| M | I | 1 | *COL1A1* | intron 26 | Splice +1G>A | c.1821+1G>A | 0 | Splice site |
| M | I | 0 | *COL1A1* | intron 34 | Splice +1G>A | c.2343+1G>A | 0 | Splice site |
| M | I | 1 | *COL1A1* | intron 3 | Splice +2T>C | c.333+2T>C | 0 | Splice site |
| M | I | 0 | *COL1A1* | 37 | p.(Pro850Leufs*258) | c.2549delC | 0 | Frameshift |
| M | I | 1 | *COL1A1* | 17 | p.(Gly377Trpfs*15) | c.1127dupC | 0 | Frameshift |
| M | I | 0 | *COL1A1* | 21 | p.(Pro460Leufs*81) | c.1379delC | 0 | Frameshift |
| M | I | 0 | *COL1A1* | 21 | p.(Pro460Leufs*81) | c.1379delC | 0 | Frameshift |
| M | I | 0 | *COL1A1* | 12 | p.(Gly281Ser) | c.841G>A | 1 | Missense |
| M | I | 1 | *COL1A1* | 7 | p.(Gly194Valfs*71) | c.579delT | 0 | Frameshift |
| M | I | 0 | *COL1A1* | 37 | p.(Gly851Alafs*257) | c.2550delT | 0 | Frameshift |
| M | I | 0 | *COL1A1* | intron 42 | Splice +1G>A | c.3045+1G>A | 0 | Splice site |
| M | I | 0 | *COL1A1* | 41 | p.(Val961Serfs*147) | c.2881delG | 0 | Frameshift |
| M | I | 1 | *COL1A1* | 48 | p.(Gly1169Ser) | c.3505G>A | 1 | Missense |
| M | I | 1 | *COL1A1* | 15 | p.(Ala327*) | c.972_978dup | 0 | Nonsense |
| M | I | 0 | *COL1A1* | 45 | p.(Val1078Alafs*29) | c.3233_3236del | 0 | Frameshift |
| M | I | 0 | *COL1A1* | 23 |  | c.1601T>A + c.1608_1614+25del | 0 | Deletion |
| M | I | 0 | *COL1A1* | 7 | p.(Gly194Valfs*71) | c.579delT | 0 | Frameshift |
| M | I | 1 | *COL1A1* | 12 | p.(Gly284Ala) | c.851G>C | 1 | Missense |
| M | I | 0 | *COL1A1* | 17 | p.(Ser363Cysfs*4) | c.1086_1102del | 0 | Frameshift |
| M | I | 0 | *COL1A1* | 52 | p.(Asp1446Glyfs*105) | c.4332dupC | 0 | Frameshift |
| M | I | 0 | *COL1A1* | 23 | p.(Lys538Argfs*3) | c.1611delC | 0 | Frameshift |
| M | I | 0 | *COL1A1* | 26 | p.(Arg598*) | c.1792C>T | 0 | Nonsense |
| M | I | 0 | *COL1A1* | 50 | p.(Gly1272Val) | c.3815G>T | 1 | Missense |
| M | I | 1 | *COL1A1* | 5 | p.(Gly154Trpfs*15) | c.458dupC | 0 | Frameshift |
| M | I | 0 | *COL1A1* | 7 | p.(Gly194Valfs*71) | c.579delT | 0 | Frameshift |
| M | I | 1 | *COL1A1* | 8 | p.(Gly197Cys) | c.589G>T | 1 | Missense |
| M | I | 0 | *COL1A1* | 17 | p.(Gly377Trpfs*15) | c.1127dupC | 0 | Frameshift |
| M | I | 0 | *COL1A1* | intron 20 | Splice -12G>A | c.1354-12G>A | 0 | Splice site |
| M | I | 1 | *COL1A1* | 42 | p.(Pro1009Leufs*99) | c.3026delC | 0 | Frameshift |
| M | I | 0 | *COL1A1* | 7 | p.(Gly188Asp) | c.563G>A | 1 | Missense |
| M | I | 0 | *COL1A1* | 19 | p.(Arg415*) | c.1243C>T | 0 | Nonsense |
| F | I | 1 | *COL1A1* | 15 | p.(Gly326Asp) | c.977G>A | 1 | Missense |
| F | I | 0 | *COL1A1* | 9 | p.(Arg220*) | c.658C>T | 0 | Nonsense |
| F | I | 1 | *COL1A1* | 48 | p.(Gly1169Ser) | c.3505G>A | 1 | Missense |
| F | I | 1 | *COL1A1* | 52 | p.(Phe1463Serfs*63) | c.4386delC | 0 | Frameshift |
| F | I | 0 | *COL1A1* | intron 39 | - | c.2667+3_2667+6del | 0 | Deletion |
| F | I | 1 | *COL1A1* | 49 | p.[Met1264Val;Met1264Glufs*59] | c.3790A>G | 1 | Frameshift |
| F | I | 0 | *COL1A1* | 8 | p.(Gly197Cys) | c.589G>T | 1 | Missense |
| F | I | 0 | *COL1A1* | intron 39 | Splice - 1G>A | c.2668-1G>A | 0 | Splice site |
| F | I | 0 | *COL1A1* | 43 | Gly1028indel | c.3091-?_indel? | 0 | Deletion |
| F | I | 1 | *COL1A1* | 37 | p.(Gly842Alafs*266) | c.2525delG | 0 | Frameshift |
| F | I | 0 | *COL1A1* | 31 | p.(Ala699Glnfs*10) | c.2091_2092del | 0 | Frameshift |
| F | I | 0 | *COL1A1* | 2 | p.(Ile38Alafs*34) | c.111_117del | 0 | Frameshift |
| F | I | 0 | *COL1A1* | 12 | p.(Gly284Ala) | c.851G>C | 1 | Missense |
| F | I | 1 | *COL1A1* | 5 | p.(Gly154Trpfs*15) | c.458dupC | 0 | Frameshift |
| F | I | 0 | *COL1A1* | 36 | p.(Gly809Argfs*12) | c.2424dupC | 0 | Frameshift |
| F | I | 1 | *COL1A1* | intron 47 | Splice -5G>C | c.3424-6C>G | 0 | Splice site |
| F | I | 0 | *COL1A1* | 19 | p.(Gln421Argfs*120)^1^ | c.1261delC^1^ | 0 | Frameshift |
| F | I | 0 | *COL1A1* | 5 | p.(Gly145Argfs*24) | c.432dupC | 0 | Frameshift |
| F | I | 0 | *COL1A1* | 25 | p.(Gly560Ser) | c.1678G>A | 1 | Missense |
| M | I | 0 | *COL1A1* | intron 3 | Splice +2 T>C | c.333+2T>C | 0 | Splice site |
| F | I | 0 | *COL1A1* | intron 26 | Splice -1 G>A | c.1821+1G>A | 0 | Splice site |
| M | I | 1 | *COL1A2* | 31 | p.(Gly601Ser) | c.1801G>A | 1 | Missense |
| M | I | 1 | *COL1A2* | 19 | p.(Gly337Ser) | c.1009G>A | 1 | Missense |
| M | I | 0 | *COL1A2* | 12 | p.(Gly193Ser) | c.577G>A | 1 | Missense |
| M | I | 0 | *COL1A2* | 12 | p.(Gly193Ser) | c.577G>A | 1 | Missense |
| M | I | 0 | *COL1A2* | 12 | p.(Gly193Ser) | c.577G>A | 1 | Missense |
| M | I | 0 | *COL1A2* | 8 | p.(Gly109Asp) | c.326G>A | 1 | Missense |
| M | I | 0 | *COL1A2* | 12 | p.(Gly193Ser) | c.577G>A | 1 | Missense |
| F | I | 1 | *COL1A2* | intron 43 | Splice +1 G>A | c.2835+1G>A | 1 | Splice site |
| F | I | 1 | *COL1A2* | 19 | p.(Gly337Ser) | c.1009G>A | 1 | Missense |
| F | I | 0 | *COL1A2* | 49 | p.(Gly1102Cys) | c.3304G>T | 1 | Missense |
| F | I | 1 | *COL1A2* | intron 21 | Splice +5G>A | c.1197+5G>A | 1 | Splice site |
| F | I | 1 | *COL1A2* | 17 | p.(Gly265Arg) | c.793G>C | 1 | Missense |
| F | I | 1 | *COL1A2* | 17 | p.(Gly286Ser) | c.856G>A | 1 | Missense |
| M | I | 0 | *COL1A1* | Intron 19 | Splice +1G>A | c.1299+1G>A | 0 | Splice site |
| M | I | 1 | *COL1A1* | 11 | p.(Gly251Asp) | c.752G>A | 1 | Missense |
| M | I | 1 | *COL1A1* | 25 | p.(Gly560Ser) | c.1678G>A | 1 | Missense |
| M | I | 0 | *COL1A1* | Intron 15 | Splice +2T>C | c.1002+2T>C | 0 | Splice site |
| F | I | 1 | *COL1A1* | 49 | p.(Gly1190Valfs*49) | c.3567delT | 0 | Frameshift |
| F | I | 0 | *COL1A1* | 41 | p.(Ser979Leufs*129) | c.2934delC | 0 | Frameshift |
| M | I | I | *COL1A1* | 45 | p.(Gly1079Ser) | c.3235G>A | 1 | Missense |
| M | I | 0 | *COL1A1* | 49 | p.(Lys1263Argfs*68) | c.3788delA | 0 | Frameshift |
| M | I | 0 | *COL1A1* | x | Large deletion of  *COL1A1* | - | 0 | Deletion |
| F | I | 0 | *COL1A1* | 31 | p.(Arg697*) | c.2089C>T | 0 | Nonsense |
| F | I | 0 | *COL1A1* | 5 | p.(Gly145Argfs*24) | c.432dupC | 0 | Frameshift |
| F | I | 0 | *COL1A1* | 49 | p.(Ser1251Argfs*82) | c.3748_3752dup | 0 | Frameshift |
| M | I | 0 | *COL1A1* | 17 | p.(Gly377Trpfs*15) | c.1127dupC | 0 | Frameshift |
| M | I | 0 | *COL1A1* | 17 | p.(Arg361*) | c.1081C>T | 0 | Nonsense |
| F | I | 0 | *COL1A1* | 49 | p.(Gln1203*) | c.3607C>T | 0 | Nonsense |
| M | I | 0 | *COL1A1* | 11 | p.(Gly257Arg)^2^ | c.769G>A ^2^ | 1 | Missense |
| F | I | 0 | Unknown | x | x | x | x | Unknown |
| F | I | 0 | Unknown | x | x | x | x | Unknown |
| M | I | 1 | Unknown | x | x | x | x | Unknown |
| M | I | 0 | Unknown | x | x | x | x | Unknown |
| M | I | 0 | Unknown | x | x | x | x | Unknown |
| M | I | 0 | Unknown | x | x | x | x | Unknown |
| M | I | 0 | Unknown | x | x | x | x | Unknown |
| M | I | 0 | Unknown | x | x | x | x | Unknown |
| M | I | 0 | Unknown | x | x | x | x | Unknown |
| F | I | 0 | Unknown | x | x | x | x | Unknown |
| M | I | 0 | Unknown | x | x | x | x | Unknown |
| M | I | 0 | Unknown | x | x | x | x | Unknown |
| F | I | 0 | Unknown | x | x | x | x | Unknown |
| F | III | 1 | *COL1A1* | 38 | p.(Gly866Ser) | c.2596G>A | 1 | Missense |
| M | III | 1 | *COL1A1* | Intron 32 | Splice +1 G>A | c.2235+1G>A | 1 | Splice site |
| M | III | 1 | *COL1A1* | 45 | p.(Gly1076Ser) | c.3226G>A | 1 | Missense |
| M | III | 1 | *COL1A1* | 44 | p.(Gly1040Ser) | c.3118G>A | 1 | Missense |
| F | III | 1 | *COL1A2* | 44 | p.Gly973Val | c.2918G>T | 1 | Missense |
| F | III | 1 | *COL1A2* | 23 | p.Arg423His | c.1268G>A | 1 | Missense |
| F | III | 1 | *COL1A2* | 32 | p.Gly646Val | c.1937G>T | 1 | Missense |
| F | III | 1 | *COL1A1* | 44 | p.(Gly1040Ser) | c.3118G>A | 1 | Missense |
| M | III | 1 | *COL1A1* | Intron 44 | Splice -6C>T^2^ | c.3208-6C>T^2^ | ? | Splice site |
| F | III | 1 | *COL1A1* | 31 | p.(Gly692Ala) | c.2075G>C | 1 | Missense |
| F | III | 1 | *COL1A2* | 46 | p.(Gly1003Asp) | c.3008G>A | 1 | Missense |
| F | III | 1 | *COL1A2* | 21 | p.(Gly388Arg) | c.1162G>C | 1 | Missense |
| F | III | 1 | *COL1A2* | 19 | p.Gly331Asp^1^ | c.992G>A^1^ | 1 | Missense |
| M | III | 1 | *COL1A1* | Intron 32 | Splice +1G>A | c.2235+1G>A | 1 | Splice site |
| F | III | 1 | *COL1A1* | 32 | p.(Gly719Ser) | c.2155G>A | 1 | Missense |
| F | III | 0 | *COL1A1* | 1 | p.(Gly22_Gln23del) | c.65_70del | 1 | Deletion |
| M | III | 1 | *COL1A2* | 46 | p.(Gly1030Ala) | c.3089G>C | 1 | Missense |
| F | III | 1 | *COL1A2* | x | Large deletion of *COL1A2* | - | 0 | Deletion |
| F | III | 0 | Unknown | x | x | x | x | Unknown |
| M | III | 1 | Unknown | x | x | x | x | Unknown |
| F | III | 0 | Unknown | x | x | x | x | Unknown |
| F | III | 1 | Unknown | x | x | x | x | Unknown |
| M | IV | 1 | *COL1A1* | 19 | p.(Gly401Ser) | c.1201G>A | 1 | Missense |
| M | IV | 1 | *COL1A1* | 15 | p.(Gly332Arg) | c.994G>A | 1 | Missense |
| M | IV | 1 | *COL1A1* | 37 | p.(Gly821Ser) | c.2461G>A | 1 | Missense |
| F | IV | 0 | *COL1A1* | 51 | p.(Asp1413Glu) | c.4239T>A | 1 | Missense |
| F | IV | 1 | *COL1A1* | 37 | p.(Gly839Ser) | c.2515G>A | 1 | Missense |
| F | IV | 1 | *COL1A1* | 31 | p.(Arg697*) | c.2089C>T | 0 | Nonsense |
| F | IV | 1 | *COL1A1* | 17 | p.(Gly353Ser) | c.1057G>A | 1 | Missense |
| F | IV | 0 | *COL1A1* | 49 | p.(Asp1219Asn) | c.3655G>A | 1 | Missense |
| F | IV | 0 | *COL1A1* | 50 | p.(Cys1299Trp) | c.3897C>G | 1 | Missense |
| M | IV | 1 | *COL1A2* | 46 | p.(Gly1012Ser) | c.3034G>A | 1 | Missense |
| M | IV | 1 | *COL1A2* | 25 | p.Gly469Ala | c.1406G>C | 1 | Missense |
| M | IV | 0 | *COL1A2* | intron 36 | Splice -4A>C | c.2188-4A>C, c.2717G>A | 1 | Splice site |
| F | IV | 1 | *COL1A2* | 17 | p.(Gly265Asp) | c.794G>A | 1 | Missense |
| F | IV | 1 | *COL1A2* | 21 | p.(Gly391Ser) | c.1171G>A | 1 | Missense |
| F | IV | 0 | *COL1A2* | 17 | p.(Gly292Ser) | c.874G>A | 1 | Missense |
| M | IV | I | *COL1A1* | 37 | p.(Gly821Ser) | c.2461G>A | 1 | Missense |
| F | IV | 1 | *COL1A2* | 47 | p.(Gly1036Arg) |  | 1 | Missense |
| M | IV | 1 | *COL1A1* | 31 | p.(Gly704Cys) | c.2110G>T | 1 | Missense |
| F | IV | 0 | *COL1A1* | 52 | p.(Thr1431Ile) | c.4292C>T | 1 | Missense |
| M | IV | 1 | *COL1A1* | 33_34 | p.(Gly779Ser) | c.2335G>A | 1 | Missense |
| M | IV | 1 | *COL1A1* | 50 | p.(Gly1272Val) | c.3815G>T | 1 | Missense |
| F | IV | 1 | *COL1A2* | intron 43 | Splice +1G>A | c.2835+1G>A | 1 | Splice site |
| M | IV | 0 | Unknown | x | x | x | x | Unknown |
| F | IV | 0 | Unknown | x | x | x | x | Unknown |
| M | IV | 1 | Unknown | x | x | x | x | Unknown |
| M | IV | 1 | Unknown | x | x | x | x | Unknown |
| M | IV | 1 | Unknown | x | x | x | x | Unknown |
| M | IV | 0 | Unknown | x | x | x | x | Unknown |
| M | IV | 1 | Unknown | x | x | x | x | Unknown |
| M | IV | 1 | Unknown | x | x | x | x | Unknown |
| F | IV | 1 | Unknown | x | x | x | x | Unknown |
| F | IV | 1 | Unknown | x | x | x | x | Unknown |
| F | IV | 0 | Unknown | x | x | x | x | Unknown |
| M | IV | 1 | Unknown | x | x | x | x | Unknown |

^1^Analysis performed in another laboratory

^2^ The variant in this patient is not predicted to markedly affect mRNA splicing so it is probably not disease-causing.
